# Effect of school feeding program on dietary folate intake among school adolescent girls in Sidama region, southern Ethiopia

**DOI:** 10.3389/fnut.2024.1495824

**Published:** 2024-11-06

**Authors:** Amelo Bolka, Tafese Bosha, Samson Gebremedhin

**Affiliations:** ^1^School of Nutrition, Food Science and Technology, Hawassa University, Awasa, Ethiopia; ^2^School of Public Health, Addis Ababa University, Addis Ababa, Ethiopia

**Keywords:** adolescent girls, dietary folate intake, school-feeding program, Sidama region, southern Ethiopia

## Abstract

**Background:**

Ethiopia recently initiated a school feeding program (SFP) that provides nutritious meals to vulnerable students during school to combat malnutrition and improve their nutrition. However, there is limited evidence regarding the program’s impact on dietary folate intake among adolescent girls. Improving folate status in adolescent girls is important to prevent neural tube defect and anemia This study was aimed at assessing the effect of the SFP on dietary folate intake among school adolescent girls in the Sidama Region, Southern Ethiopia.

**Methods:**

This school-based cross-sectional study compared dietary folate intake among adolescent girls enrolled in schools with (*n* = 290) and without (*n* = 290) SFP. Multistage sampling method was used to select adolescent girls from 12 schools. The multiple pass 24-h dietary recall method was used to estimate the dietary intake of adolescent girls. Dietary diversity in the preceding day of the survey was assessed with the standard nine-food group score. Nutrient inadequacy was defined as a proportion of adolescents with dietary value of nutrient intake less than the recommended daily allowance (RDA). The dietary folate intake was compared between two groups using multilevel mixed effects linear regression model adjusted for potential confounders.

**Results:**

The result showed significantly higher mean (±SD) dietary folate intake in SFP beneficiaries (421.12 ± 78.60 μg/day) than the non-beneficiaries (393.26 ± 74.57 μg/day; β = 27.19, *p* < 0.001). SFP beneficiary girls had a significantly higher mean (±SD) dietary diversity score (5.24 ± 1.35) compared to non-beneficiary girls (4.83 ± 1.54; β = 0.40, *p* = 0.001). The prevalence of inadequate folate intake was significantly higher among no-beneficiaries (47.4%) compared to beneficiaries (36.9%; *p* = 0.01).

**Conclusion:**

The SFP in Sidama region improved folate intake and dietary diversity of adolescent girls. The evidence from this study supports the expansion of the program to enhance dietary intake of nutrients of adolescent girls in the region.

## Introduction

Adolescence (10–19 years) is a period of rapid growth and development (1, 2) necessitating increased nutrient intake. Increased physiological requirements during this period means adolescents are more vulnerable to nutrient deficiencies (3). Undernutrition including micronutrient deficiencies in adolescence may hamper physical and cognitive development, cause stunting, and compromise immunity (4). Undernutrition also increases the risk of anemia, osteoporosis, and causes delayed sexual maturation (5).

School feeding program (SFP) is a targeted social safety net that provides nutritious meals to socio-economically disadvantaged school children during the school day, aiming to prevent hunger and improve their nutritional status and overall well-being (6). The program is vital for children, especially those from food insecure areas in low-income settings like Ethiopia, as they rely on these meals as their major daily sustenance. SFP has the potential to contributing to the achievement of Sustainable Development Goal (SDG) two (ending hunger) (7). The SFP provides various benefits, including alleviating short-term hunger, increasing school enrolment, reducing dropout and absenteeism rates, promoting girls’ education, and narrowing gender disparities in school enrolments, as documented by numerous studies (8–11).

Globally SFP benefits approximately 418 million children. Coverage varies based on income level, with 18% of school children in low-income countries receiving free or subsidized meals, compared to 61% in high-income countries (12). In Africa, 31% of children in primary schools, receive meals through the SFP. The coverage of SFP is higher in upper middle-income countries at 55%, while low-income countries have coverage of 15% (7). Despite the efforts, around 62.7 million schoolchildren in Africa attend school hungry (13, 14).

In Ethiopia, SFP was first launched in 1994, Tigray region (15). The program gradually expanded to cover most regions, including Addis Ababa City Administration (16). School feeding program is a vital nutrition-sensitive initiative aimed at combating malnutrition through promoting local agriculture and ensuring equitable education access (17, 18). The program implementation in schools resulted in enhanced academic achievement and improved enrolment (11, 19), and improved dietary diversity and nutritional status of beneficiaries (20).

Folate is a critical nutrient during adolescence, as it plays a crucial role in cell division, DNA synthesis, red blood cell formation, and development (21). Adequate intake supports cognitive function, emotional well-being, and rapid growth (22). Increased needs during puberty can be met through dietary sources like cereals, legumes, leafy vegetables, fruits, liver, egg yolks, and yeast. However, many adolescent girls often lack sufficient folate-rich foods in their diet, leading to deficiencies.

Folate deficiency causes significant long-term health problems. In adolescents, it leads to anemia, impaired cognitive and immune function, and developmental delays (23, 24). Pregnant women with low folate levels face increased risks of pregnancy complications (abruptio placentae, preeclampsia, miscarriage, stillbirth, preterm delivery, low birth weight) and serious congenital anomalies of the brain and spine, such as neural tube defects, underscoring the need for targeted nutritional strategies to ensure sufficient folate levels in this critical growth period (21, 25).

School feeding program in low-income countries including Ethiopia are targeted at preventing hunger in students. However, school meals should also provide school children with reasons nutrient density. Adequate nutrients intake among school adolescent girls is a critical issue requiring immediate attention due to their association with health problems (26). Many studies have been conducted to assess the effects of SFP on health (27, 28), educational outcomes (11, 19, 29) and anthropometric status (20, 30). However, the effect of the program on folate status of adolescent girls has not been assessed so far. Thus, this study was aimed at assessing the effect of the school feeding program on dietary folate intake among school adolescent girls in Sidama Region, Southern Ethiopia.

## Materials and methods

### Study setting

Sidama Region is one of the 12 regional states of Ethiopia with a population size of nearly 5 million. The altitude of the region ranges from 500 to 3,500 m above sea level. More than 85% of the population livelihood in the region depends on the farming, with a predominant focus on mixed crop-livestock production. Major crops grown in the region include *enset* (false banana), maize, wheat, barley and teff, and root and tuber crops. The coffee and khat are cash crops in the region. The region has 30 rural districts, 6 town administrations, and a city administration (31).

The school feeding program in Sidama has been implemented in Boricha district, serving as the sole district in the region to benefit from this initiative. Within the district, there were twenty-one first and second cycle schools covered by the program. A total of 13,831 adolescent girls were enrolled in the program during the study period. Every enrolled adolescent girl received a daily one hot meal. According to the program standard, each SFP beneficiary adolescent girl receives a 150 g meal made from wheat, corn, soybean, whole lentil, or vegetable oil once daily from Monday to Friday (31).

### Source and study population

All school adolescent girls, age within 10–19 years, with and without SFP in Sidama region Randomly selected school adolescent girls (10–19 years) regularly enrolled in schools with and without school feeding program in the region were the population of the study.

### Inclusion and exclusion criteria

School-enrolled adolescent girls in Sidama region willing to participate in the study were included. However, adolescent girls who were pregnant or in the postpartum period during the study or with physical and mental disabilities were excluded.

### Sample size determination

The sample size to compare the folate intake between adolescent girls enrolled in schools with and without SFP was calculated using the G*Power 3.1.9.4 program (32). The assumptions taken into account were: 95% confidence level, a margin of error of 5, 80% power, a one-to-one allocation ratio between the two groups, and a medium effect size (d = 0.3) and design effect of 1.5. The non-response rate of 10% was regarded as a probable dropout composite. In the end, a sample size of 580 participants—290 school feeding program beneficiaries and 290 non-beneficiaries—was considered sufficient.

### Sampling procedure

This study used multistage sampling techniques. Boricha district was selected for its exclusive program implementation in the region. Among the 21 primary and junior schools in the district, six schools were selected using a simple random sampling method. Among districts that were not implementing the feeding program, two districts (Darara and Bilate Zuria) were purposively selected using pre-defined matching criteria. The criteria included (1): comparable agro-ecological characteristics (2), proximity to the SFP-implementing district, and (3) comparable socio-economic indicators (wealth index and household food security). Consequently, six SFP non-beneficiary schools (three from each district) were included. Adolescent girls were chosen using the same method based on school lists. The sample size was allocated proportionally to schools based on the number of girls in each.

### Study variables

The exposure of interest was SFP enrollment status, categorized as beneficiary (enrolled in SFP school) or non-beneficiary (enrolled in non-SFP school). The primary outcome was dietary folate intake level, with dietary diversity score as a secondary outcome. Dietary inadequacy was defined as intake of folate below the recommended daily allowance (DRA) of 400 μg/day, otherwise considered adequate intake (33).

Adolescent girls who consumed five or more food groups out of nine (starchy staples, legumes, dark green leafy vegetables, other vitamin A rich fruits and vegetables, other fruits and vegetables, organ meat, meat and fish, eggs, and milk and milk products) were considered to have a high dietary diversity score. This score was calculated by summing the number of distinct food groups consumed within a 24-h recall period. Scores below five indicated a lower level of dietary diversity (34).

Body mass index-for-age z-score (BAZ) and height-for-age z-score (HAZ) were calculated based on the 2007 WHO reference values. Thinness was defined as a BAZ score below −2 standard deviations. Stunting referred to a HAZ score below −2 standard deviations (35).

Factors considered as potential confounders included: age of the adolescent girl, mother’s education status, father’s education status, mother’s occupation, father’s occupation, family size, place of residence, wealth index, food security, enrollment in safety net program, nutrition education, folate knowledge, and meal frequency.

### Data collection and measurements

Data were collected using a structured and pretested interviewer-administered questionnaire developed based on review of relevant literature (11, 36–38). The questionnaire covered socio-demographic and economic characteristics, household food security, dietary practices, sanitation and hygiene practice, and anthropometric measurements. Data collection involved five data collectors and two supervisors, who received 5 days of training on the KoboToolbox system and 24-h recall interview skills. The data collectors conducted face-to-face interviews and recorded the data using the KoboToolbox application installed on Android devices. Rigorous supervision included daily examinations and prompt error corrections. The collected data were submitted to a central server.

### Dietary intake assessment

Multiple pass 24-h dietary recall method was used to assess the dietary intake of adolescent girls. In the method, four passes were followed to collect detailed information on food type and quantity. In the first pass, respondents recalled all the foods and beverages consumed in the previous 24 h. The second pass involved detailed descriptions of food preparation, including cooking methods, as well as the time and place of consumption. In the next pass, portion sizes and amounts of each food and beverage consumed were probed using standardized measurement methods. Finally, the entire list of data was reviewed to ensure completeness, accuracy and identify errors.

Dietary diversity was assessed using a 24-h recall questionnaire provided by the Food and Agriculture Organization (FAO). Respondents reported their food consumption from 16 predefined categories within the past 24 h, both at home and away. The Women’s Dietary Diversity Score (WDDS) was calculated by summing the number of food groups consumed from nine categories over a 24-h period: starchy staples, dark green leafy vegetables, other vitamin A-rich vegetables and fruits, other vegetables and fruits, organ meat, meat and fish, eggs, legumes, nuts and seeds, milk and milk products (34).

### Household food security

Household food security was assessed using the Household Food Insecurity Access Scale (HFIAS), which examined nine events over the past 4 weeks. Based on the scale, households were classified as food secure if respondents answered ‘no’ to all items (1–9) and insecure if they answered ‘yes’ to at least one item (39).

### Nutritional knowledge of adolescent girls

Adolescent girls’ nutritional knowledge on folate and anemia was assessed using 12 and 14 questions, respectively. The questions aimed to determine whether girls know about folate-rich foods, the causes, consequences, and prevention methods of folate deficiency and anemia. Scores were assigned to each response, with correct answers receiving one point and incorrect answers receiving zero. A cumulative score was calculated for both domains (40, 41).

### Anthropometric measurements

Anthropometric measurements were taken following standard procedures and using calibrated equipment. To avoid inter-observer variation, anthropometric measurements were collected by a trained and experienced nutritionist. Weight was measured using a SECA digital scale (Seca GmbH & Co. KG, Germany), without shoes and with light clothing, to the nearest 100 grams. The scale was placed on a flat surface. Height was measured using a tape attached to a vertical wall, with a horizontal headboard touching the highest point of the head. Height was recorded in meters to the nearest 0.1 cm, while barefoot or in thin socks (35).

### Data management and analysis

Data were collected using KoboToolbox system. STATA version 14 was used for data analysis. Data were described using frequency distributions, measures of central tendency and dispersion. The household’s wealth index, a metrics of household living standard, was calculated using principal component analysis (PCA) based on the ownership of valuable assets, housing conditions and access to social services.

Portion sizes from the 24-h recall were manually converted into weights (in grams). Nutritional values per 100 grams were determined using the Ethiopian food composition tables (EFCT). For food items not covered by the EFCT, relevant African countries’ food composition data were utilized (42, 43).

Adolescent girls’ nutrient intake adequacy was assessed by comparing their nutrient intake to the recommended dietary allowance (RDA). Adolescent girls’ intake below the RDA indicated inadequate dietary intake. Dietary diversity was measured using FAO’s standardized tool for women’s dietary diversity (44).

Chi-square or independent t-tests were employed to compare two groups’ socio-demographic/economic characteristics, depending on the variable’s nature. The mean difference in dietary folate intake level, and dietary diversity score between the SFP beneficiaries and non-beneficiaries was compared using an independent sample t-test. Multilevel mixed effects linear regression model was performed to determine the effect of the SFP on dietary folate intake level, and dietary diversity score. The model assumptions (linearity, multicollinearity, normality, and homoscedasticity) were checked and verified (45, 46). Akaike Information Criteria (AIC) and Bayesian Information Criteria (BIC) were used to assess model goodness of fit. In the model, a random intercept was fitted for schools. Multicollinearity was examined using the variance inflation factor (VIF). Statistical significance in the multivariable analysis was declared at a *p*-value <0.05.

Anthropometric indices, specifically HAZ and BAZ were generated using the WHO Anthro Plus software based on the WHO 2007 growth reference data. A BAZ-score below −2 was defined as thin, and a HAZ-score below −2 was defined as stunted.

## Results

### Socio-demographic characteristics

In this study, 290 adolescent girls from SFP beneficiary schools and 289 from non-SFP beneficiary schools were included, with a response rate of 99.5%. The mean (±SD) age of SFP beneficiary and non-beneficiary girls was 13.1 ± 0.1 and 13.4 ± 0.5 years, respectively. Most of the girls were in early adolescent age (10–14 years) in both non-beneficiary (67.5%) and beneficiary (72.7%) schools. The vast majority (95.8% beneficiary and 85.1% non-beneficiary) of the girls were Sidama in ethnicity. More than four-fifths of respondents, 87.2% of beneficiaries and 88.2% of non-beneficiaries, identified themselves as Protestant Christians.

Most of the mothers of the index girls were housewives in both beneficiary (77.2%) and non-beneficiary (80.3%) schools. The majority of their fathers were farmers (78.6% beneficiary, 80% non-beneficiary). The mean (±SD) family size of the households was 5.7 ± 0.1 in beneficiary and 5.8 ± 0.1 in non-beneficiary groups. The majority of girls families resided in rural areas (78.6 beneficiary and 82.7% non-beneficiary). Fewer than one-fifth (17.6%) of families of adolescent girls from both groups were enrolled in the productive safety net program.

Statistically significant differences were observed in mother’s education, father’s education, and wealth index between the SFP beneficiary and non-beneficiary groups. SFP beneficiary adolescent girls had significantly better maternal and paternal educational statuses compared to their counterparts (*p* < 0.001). SFP beneficiaries were also economically advantaged based on the wealth index (*p* < 0.05; Table 1).

**Table 1:** Socio-demographic and economic characteristics of adolescent girls in Sidama Region, 1 January to 28 March 2024.

Variables	School Feeding Program		*p*-value^x^
	Beneficiaries (*n* = 290)	Non-beneficiaries (*n* = 289)	
Mean age (±SD) of girls	13.11 ± 2.33 years	12.91 ± 2.35 years	0.296
Mean age (±SD) of mothers	36.67 ± 6.03 years	36.69 ± 6.77 years	0.967
Ethnic group (%)			
Sidama	91	87.9	0.218
Others	9	12.1	
Religion (%)			
Protestant	82.4	86.5	0.266
Others	17.6	13.5	
Parents marital status (%)			
Married	98.9	98.3	0.473
Divorced	1.1	1.7	
Mother’s education status (%)			
No formal education	44.8	59.1	<0.001^*^
Formal education	55.2	40.9	
Father’s education status (%)			
No formal education	46.9	60.5	<0.001^*^
Formal education	53.1	39.5	
Mother’s occupation (%)			
Housewife	77.2	80.2	0.372
Other works	22.8	19.8	
Father’s occupation (%)			
Farmer	78.6	79.9	0.697
Other works	21.4	20.1	
Household wealth index (%)			
Lowest	30.3	31.8	0.02^*^
Middle	22.1	30.8	
Highest	47.6	37.4	
Mean (±SD) family size	5.7 ± 1.4	5.8 ± 1.4	0.880
Enrollment in productive safety net program (%)			
Yes	18	17.6	0.929
No	82	82.4	
Place of residence (%)			
Rural	78.6	82.7	0.214
Urban	21.4	17.3	

x Chi-square or independent *t*-test.

* Statistically significant difference at *p*-value of 0.05.

### Consumption of folate rich foods

The mean (±standard deviation) folate knowledge of girls was 3.8 ± 1.2 for SFP beneficiaries and 3.6 ± 1.6 for non-beneficiary girls, with no significant difference between the two groups (*p* = 0.153). The most consumed folate-rich foods in the study area were: rice, spaghetti, and bread (89.3%), legumes (peas, peanuts, and beans; 80.3%), vegetables and fruits (74.6%), and milk (59.9%). Eggs (36.4%) and meat (28.7%) were the least consumed folate-rich foods, while no one reported consuming fish as a source of folate during the same period.

### Food security, dietary diversity and meal frequency

Food insecurity was observed in 43.4% of SFP beneficiaries and 56.0% of non-beneficiary households, indicating a statistically significant difference (*p* = 0.002). SFP beneficiary girls had a significantly higher mean (±SD) dietary diversity score (5.2 ± 1.3) compared to non-beneficiary girls (4.8 ± 1.5; *p* < 0.001, d = 0.29). The mean (±SD) number of meals consumed per day among non-beneficiaries (2.5 ± 0.6) was significantly lower than that among beneficiaries (3.1 ± 0.7; *p* < 0.001, d = 0.92).

### Anthropometric status of adolescent girls

The mean (±SD) height for age z-score among non-beneficiary girls (−0.25 ± 1.04) was significantly higher than that for SFP beneficiary girls (−0.46 ± 1.07; *p* = 0.017, d = 0.20). The mean difference was −0.21 (95% CI: −0.38, −0.04) in favor of the non-beneficiary girls. No statistically significant differences were observed between the two groups in other features like mean body mass index for age z-score, stunting, and thinness.

### Dietary folate intake among adolescent girls

**Figure 1:**
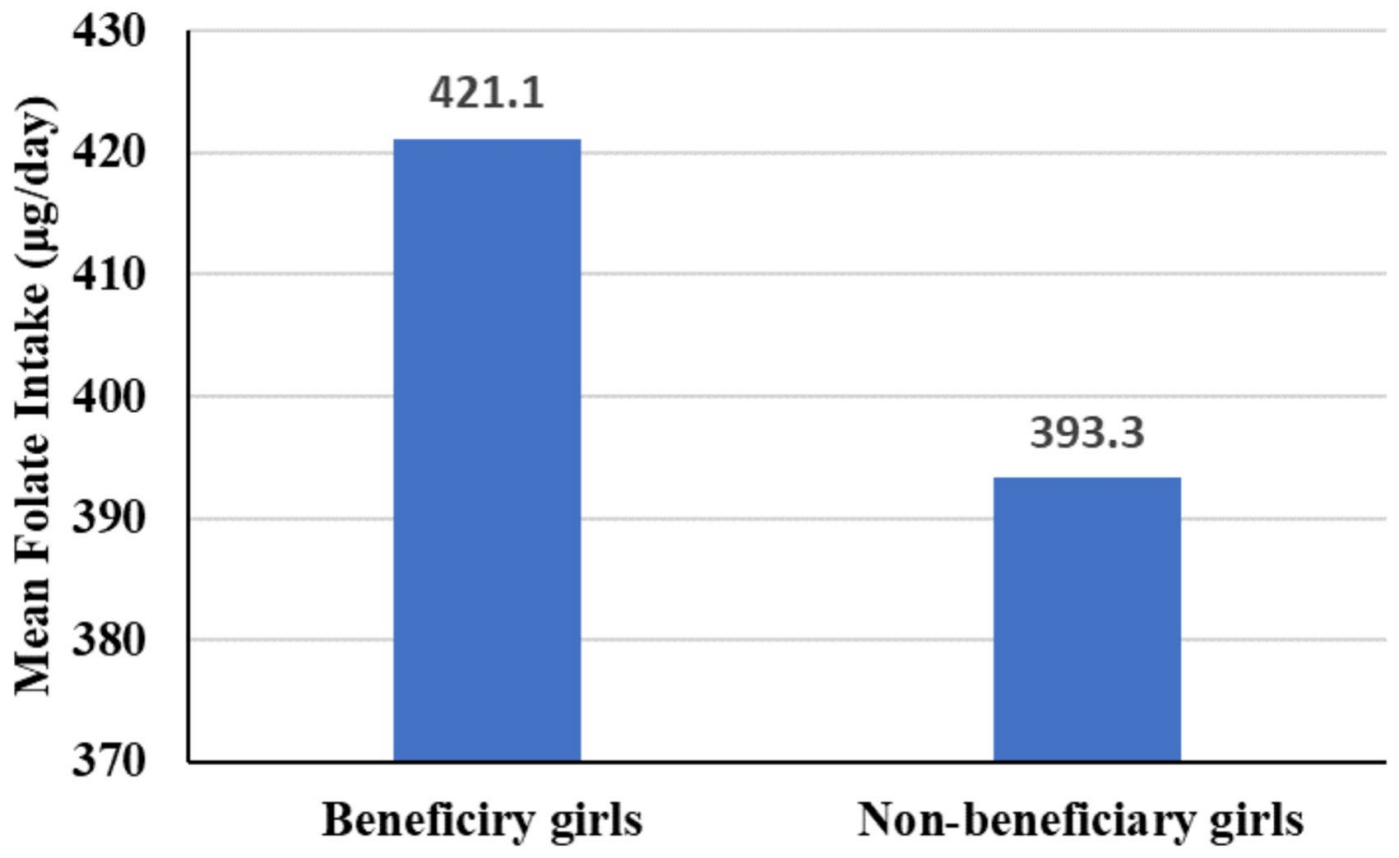
Mean dietary folate intake among beneficiary and no-beneficiary adoelscent girls in Sidama Region, 1 January to 28 March 2024.

None of the study participants reported folate intake from supplements. The mean (±SD) intake of dietary folate among beneficiary and non-beneficiary girls was 421.1 ± 78.6 μg/day and 393.3 ± 74.6 μg/day, respectively. The differences in mean dietary folate intake between the two groups were significant (*p* < 0·0001, d = 0.36), indicating an improvement in folate intake among beneficiary girls (Figure 1). The mean (±SD) dietary folate intake among beneficiary girls from school meals and home meals was 142.5 ± 26.6 μg/day and 278.6 ± 52.0 μg/day, respectively.

The consumption of a diversified diet from home meals among beneficiary and non-beneficiary girls was comparable. Among beneficiary girls, school meals contributed 35.6% and home meals contributed 69.6% of their dietary folate intake. Regarding folate needs, 39.7% of beneficiary girls and 47.2% of non-beneficiary girls did not meet daily requirements.

### Prevalence of inadequate dietary folate intake

The overall prevalence of folate intake inadequacy among adolescent girls was 42.1% (95% CI: 38.1, 46.2%). The inadequate intake of dietary folate among beneficiary and non-beneficiary adolescent girls was 36.9 and 47.4%, respectively. A significantly higher prevalence of dietary folate intake inadequacy was observed among non-beneficiary girls (*p* = 0.01).

### Effect of SFP on dietary folate intake and dietary diversity score

The effect of SFP on dietary folate intake, and dietary diversity score was assessed using mixed effects linear regression models. The results demonstrated significant positive effects of SFP on dietary folate intake level (β = 27.19, *p* < 0.001), and dietary diversity score (β = 0.40, *p* = 0.001), after adjusting for confounders (Table 2).

**Table 2:** Effect of SFP on dietary folate intake, DDS and the number of meals consumed per a day among adolescent girls in Sidama Region, 1 January to 28 March 2024.

Variable	Bivariable analysis		Multivariable analysis	
	β-coefficient	*p*-value	β-coefficient	*p*-value
Dietary folate intake	27.86	<0.001	27.19	<0.001
Dietary diversity score	0.41	<0.001	0.40	0.001

#### Variables adjusted in the model

Girls’ age, mother education status, father education status, father occupation, family size, wealth index, food security, enrollment in safety net program, nutrition education, meal frequency and folate knowledge.

## Discussion

A school-based comparative cross-sectional study was conducted in the Sidama region to assess the effects of the SFP on dietary folate intake and dietary diversity score among adolescent girls. The study found that 42.1% of the surveyed adolescent girls had inadequate dietary folate intake. The inadequate intake of dietary folate among beneficiary and non-beneficiary adolescent girls was 36.9 and 47.4%, respectively. The program significantly improved dietary folate intake and dietary diversity of the enrolled girls.

The prevalence of inadequate dietary folate intake among adolescent girls was 42.1%. Studies conducted in southcentral Ethiopia (47), northern Ethiopia (36) and Indonesia (48) reported even a higher prevalence of inadequate folate intake among adolescent girls. The high folate inadequacy could be due to low nutrition knowledge among girls, resulting in insufficient consumption of folate-rich foods (meat, fish, milk, eggs, vegetables, and fruits). Food insecurity and limited purchasing power of lower-middle wealth families also hinder access to a diverse folate-rich diet.

The findings from the current study presented that the mean dietary folate intake of the beneficiary girls was significantly increased as compared with that of the non-beneficiaries. The mean dietary folate intake difference was 27.86 μg/day, favoring SFP beneficiaries. Significantly lower prevalence of inadequate folate intake was also observed among beneficiaries (36.9%) compared to non-beneficiaries (47.4%). Research in Ghana (49) and England (50–52) supports this, showing that school-fed adolescents have better dietary folate intake. This is attributed to high household food insecurity and poor diet quality in the study area, where girls without SFP had lower chances of sufficient micronutrient intake, while SFP increased their intake, including folate, through regular meals.

The findings from the current study also confirmed that the SFP had a beneficial impact on dietary diversity score among adolescent girls. The mean dietary diversity score difference was 0.41 in favor of the SFP beneficiaries. Home meal contributions to dietary diversity were comparable, but the SFP added at least two food groups (starchy staples, and legumes, seeds and nuts), increasing the mean DDS for beneficiaries compared to non-beneficiaries. Studies conducted in southern Ethiopia (20) and Ghana (49) reported consistent findings. Improved consumption of a diversified diet ensures adequate nutrient intake in adolescent girls, supporting healthy reproductive development.

Food insecurity was one of the public health problems in the study area. It was more prevalent in non-beneficiary girls (56%) compared with beneficiaries (43.4%) with statistically significant difference. Two-thirds (66.3%) of girls with inadequate folate intake and nearly three-fourths (73.8%) with dietary diversity scores below five were from food-insecure households. More than half (55.3%) of adolescent girls consumed two or fewer meals were from food insecured household. Food insecurity reduced dietary variety, limiting choices and increasing the likelihood of micronutrient deficiencies, potentially lowering folate intake in adolescent girls (53). SFP mitigate food insecurity by ensuring regular access to nutritious meals for students, addressing hunger and improving nutrition (low-income girls) and easing the burden on families by reducing the need to provide food at home.

The study used a multiple-pass 24-h recall to assess dietary folate intake from staple foods consumed in the study area. However, recall and social desirability biases could have influenced the findings. Even though the researchers excluded data on fasting and holidays, relying on a single-day recall may not accurately represent habitual intakes. Unaddressed variables may have introduced residual confounders, and non-standardized nutritional literacy tools may have led to measurement errors. The local food composition tables in developing countries may not accurately reflect the nutritional content of local foods.

## Conclusion

This study presented a beneficial effect of the school feeding program. It identified that the implementation of the program significantly improved the dietary folate intake and dietary diversity score of adolescent girls in the Sidama region. Furthermore, the number of meals consumed per day was higher among SFP beneficiary girls.

### Recommendation

Findings from this study are informative to decision makers. Sidama National Regional State Education Bureau in collaborations with interested stakeholders should expand SFP in the region to improve dietary nutrients intake of adolescent girls. The program’s effectiveness should be monitored and evaluated regularly. Moreover, a longitudinal study is recommended to assess the long-term impact of the program on the micronutrient adequacy of adolescent girls for better policy implications.

## Data Availability

The raw data supporting the conclusions of this article will be made available by the authors, without undue reservation.

## Glossary

BAZ: Body mass index for age Z-score
CI: Confidence Interval
CL: Confidence Level
DDS: Dietary Diversity Score
EFCT: Ethiopian Food Composition Table
FAO: Food and Agriculture Organization
HAZ: Height for age Z-score
HFIAS: Household Food Insecurity Access Scale
IRB: Institutional Review Board
IQR: Interquartile Range
PCA: Principal Component Analysis
RDA: Recommended Daily Allowance
SD: Standard Deviation
SDG: Sustainable Development Goal
SFP: School Feeding Program
WFP: World Food Program
WHO: World Health Organization
